# LncRNA DANCR and miR-320a suppressed osteogenic differentiation in osteoporosis by directly inhibiting the Wnt/β-catenin signaling pathway

**DOI:** 10.1038/s12276-020-0475-0

**Published:** 2020-08-11

**Authors:** Cheng-Gong Wang, Yi-He Hu, Shi-Long Su, Da Zhong

**Affiliations:** Department of Orthopaedics, Xiangya Hospital of Central South University, 410008 Changsha, Hunan Province P.R. China

**Keywords:** Cell biology, Diseases

## Abstract

Our study aimed to determine how lncRNA DANCR, miR-320a, and CTNNB1 interact with each other and regulate osteogenic differentiation in osteoporosis. qRT-PCR and western blotting were performed to determine the expression of DANCR, miR-320a, CTNNB1, and the osteoporosis- or Wnt/β-catenin pathway-related markers T-cell factor 1 (TCF-1), runt-related transcription factor 2 (RUNX2), alkaline phosphatase (ALP), osteocalcin (OCN), and osteopontin (OPN). Interactions between CTNNB1, DANCR, and miR-320a were predicted by bioinformatics approaches and validated using a luciferase assay. Osteoblastic phenotypes were evaluated by ALP staining, ALP activity assay and Alizarin Red staining. The bilateral ovariectomy method was used to establish an in vivo osteoporosis model. Bone morphological changes were examined using hematoxylin and eosin (H&E) and Alcian Blue staining. The expression levels of DANCR and miR-320a in BMSCs derived from osteoporosis patients were upregulated, whereas CTNNB1 expression was downregulated compared with that in healthy controls. Importantly, we demonstrated that miR-320a and DANCR acted independently from each other and both inhibited CTNNB1 expression, whereas the inhibitory effect was additive when miR-320a and DANCR were cooverexpressed. Moreover, we found that DANCR overexpression largely abrogated the effect of the miR-320a inhibitor on CTNNB1 expression and the Wnt/β-catenin signaling pathway in BMSCs during osteogenic differentiation. We further confirmed the results above in BMSCs derived from an osteoporosis animal model. Taken together, our findings revealed that DANCR and miR-320a regulated the Wnt/β-catenin signaling pathway during osteogenic differentiation in osteoporosis through CTNNB1 inhibition. Our results highlight the potential value of DANCR and miR-320a as promising therapeutic targets for osteoporosis treatment.

## Osteoporosis: Tiny targets to keep bones strong

Two non-coding RNAs are potential targets for reducing bone loss in post-menopausal osteoporosis. Bones are constantly being remodeled; when resorption outpaces generation of new bone, bones are weakened, causing osteoporosis and leading to decreased quality of life and injuries. Although treatments exist, they often have undesirable side effects, and new treatments are needed. The molecular basis of the changes that accompany osteoporosis are poorly understood. Da Zhong at the Xiangya Hospital of Central South University in Changsha, China, and co-workers investigated how two non-coding RNAs, small molecules that regulate gene expression, are involved in the progression of post-menopausal osteoporosis. They found that levels of both molecules are increased in osteoporosis, and that silencing them increases building of new bone, key to maintaining bone strength. These results illuminate a potential new direction in treatments for osteoporosis.

## Introduction

Osteoporosis is a common, age-related and costly bone disease that is characterized by low bone mineral density (BMD) and impaired bone microarchitecture. Patients have greater risks of bone fractures, and in severe cases, an increased mortality rate. More than 8.9 million fractures per year are caused by osteoporosis worldwide^1^, with 16% of women and 2% of men over 50 years old being affected^2,3^. Although numerous factors, including age, nutrition, and medication use, may contribute to osteoporosis development^4^, postmenopausal osteoporosis represents the most common type of osteoporosis, and up to 50% of elderly women worldwide are estimated to suffer from estrogen-related bone fragility, which greatly impairs their quality of life^5^. A number of clinical therapeutics exist to treat osteoporosis, including bisphosphonates, selective estrogen receptor modulators (SERMs), parathyroid hormone, and hormone replacement therapy (HRT), mostly aiming at symptom management^6,7^. Despite the remarkable advances in osteoporosis drug discovery, the currently available therapeutic methods are limited by their side effects and long-term safety^8,9^. There remains an essential clinical need to develop more effective and curative approaches by obtaining a better understanding of disease progression at the molecular level.

The basic pathophysiological change in osteoporosis development is the disrupted remodeling process during bone generation, in which the balance between the formation of new bone by osteoblasts and the resorption of old bone by osteoclasts is disrupted^10^. Previous studies have shown that the Wnt/β-catenin signaling pathway (also known as the canonical Wnt pathway) acts as the master regulator of these changes by inducing osteoblast differentiation and suppressing osteoclast generation^11,12^. Bone regeneration is accelerated by activation of the Wnt/β-catenin signaling pathway^13^. Aberrant expression of CTNNB1, which is the gene that encodes β-catenin, is a frequent cause of altered Wnt signaling and has been reported to be strongly associated with a wide spectrum of cancers^14^ as well as osteoporosis^15,16^. Bone anabolic therapy targeting the Wnt/β-catenin pathway, particularly CTNNB1, represents a novel antiresorptive strategy for osteoporosis treatment^17^.

Long noncoding RNAs (lncRNAs) are a type of RNA that usually contain 200 or more nucleotides. Although lncRNAs were originally believed to be transcriptional byproducts without biological functions, recent studies have reported that they play fundamental roles in the modulation of chromatin remodeling and transcriptional and post-transcriptional regulation^18–20^. Dysregulated lncRNAs have been implicated in diverse human diseases, including osteoporosis^21,22^. The present study focused on differentiation antagonizing nonprotein coding RNA (DANCR), which was reported to be overexpressed in human circulating monocytes and plays an important role in osteoporosis^23^. However, the cause of DANCR upregulation in osteoporosis patients remains elusive and needs to be further investigated.

MicroRNAs (miRNAs) are a class of short noncoding RNAs that usually include 20–22 nucleotides. MiRNAs exert their gene regulatory function by pairing to the 3’ untranslated regions (UTRs) of the target mRNAs. Previous miRNA profiling identified that miR-320a was overexpressed in osteoporotic samples^24^. In addition, miR-320a was reported to directly target CTNNB1 in human colon cancer^25^. In the present study, we used bioinformatics tools and determined that, interestingly, DANCR also had binding sites with CTNNB1. However, the role of the interactions between DANCR, CTNNB1 and miR-320a in osteoporosis has not been investigated or reported.

Since the possible causes of osteoporosis are diverse and the underlying molecular mechanisms may be greatly different, we aimed to focus on postmenopausal osteoporosis instead of attempting to cover all osteoporosis types in the present study. We examined the expression levels of DANCR, miR-320a and CTNNB1 in human bone marrow mesenchymal stem cells (BMSCs) derived from postmenopausal osteoporosis patients and demonstrated that miR-320a directly targeted CTNNB1 and inhibited the osteogenic differentiation of BMSCs through the Wnt/β-catenin signaling pathway. Additionally, our results showed that DANCR also bound to CTNNB1 and abolished the effect of miR-320a silencing on osteogenic differentiation in BMSCs. Our findings highlight for the first time the role of the interactions between DANCR, miR-320a, and CTNNB1 in osteogenic differentiation and may aid the development of novel therapies for osteoporosis.

## Materials and methods

### Isolation and culture of human BMSCs

Human BMSCs were collected and cultured as previously described^26^. The procedures in the present study were approved by the Medical Ethics Committee of the Xiangya Hospital, Central South University (Changsha, Hunan, China) and performed in strict accordance with the relevant guidelines. Materials were obtained from the participants with written informed consent. Bone marrow samples were obtained from 30 postmenopausal women with osteoporosis and 20 postmenopausal women without osteoporosis (as a negative control) and were passed through a 70-μm nylon mesh. Mononuclear cells were separated using the Ficoll–Hypaque gradient centrifugation method (Sigma, St Louis, MO, USA) according to the manufacturer’s instructions. The isolated BMSCs were cultured in Dulbecco’s modified Eagle’s medium (DMEM) supplemented with 10% fetal bovine serum (FBS, Gibco, Grand Island, NY, USA), 100 U/mL penicillin, and 0.1 mg/mL streptomycin (Sigma). The plates were maintained at 37 °C in a humidified environment containing 5% CO_2_.

### Human BMSC osteogenic differentiation induction

Osteogenic differentiation assays were performed using isolated BMSCs as described^27^. Briefly, BMSCs were seeded at a density of 1 × 10^5^ cells/well in a 6-well plate and grown to 80% confluence. To induce osteogenic differentiation, BMSCs were cultured in osteogenic differentiation induction medium containing 200 μM ascorbic acid, 10 mM β-glycerophosphate, and 100 nM dexamethasone (Sigma) for 14 days, and the medium was changed every 3 days.

### Cell transfection

Chemically modified miR-320a mimics or miR-320a inhibitor were designed and synthesized by GenePharma (Shanghai, China) to increase the level of miR-320a or inhibit the function of miR-320a, respectively. The sequences of miR-320a mimics, miR-320a inhibitor and their NC were as follows: miR-320a mimics: sense, 5’-AAAAGCUGGGUUGAGAGGGCGA-3’ and antisense, 5’-GCCCUCUCAACCCAGCUUUUUU-3’; mimics NC: sense, 5’-UUCUCCGAACGUGUCACGUTT-3’ and antisense, 5’-ACGUGACACGUUCGGAGAATT-3’; miR-320a inhibitor: 5’-UCGCCCUCUCAACCCAGCUUUU-3’; and inhibitor NC: 5’-CAGUACUUUUGUGUAGUACAA-3’. Lentiviral expression vectors encoding the miR-320a mimics, inhibitor or their negative controls were generated as previously described^28^. Briefly, 100 nM miR-320a mimics or inhibitor was annealed and ligated into the pLKD-CMV-G lentiviral vector (Shanghai Neuron Biotech Co., Ltd., Shanghai, China), which contained a puromycin marker and a U6 Pol III promoter. 293 T cells in the logarithmic growth phase were seeded in a 10 cm plate and transfected at 60% confluence with recombinant lentiviral vector-Lipofectamine 2000 complex (Invitrogen, Carlsbad, CA, USA). The cell supernatant was collected 72 h after transfection, and the virus titer was determined. BMSCs were plated in a 6-well plate at a density of 5 × 10^4^ cells/mL. When the cells completely adhered to the wall, they were infected with 40 µL recombinant lentivirus carrying miR-320a mimics or inhibitor at a multiplicity of infection (MOI) of 5–10 in the presence of 8 μg/mL polybrene. Empty lentivirus was used as a negative control. After incubation at 37 °C for 72 h, cells were re-plated with fresh medium supplemented with 5 μg/mL puromycin.

To silence or overexpress DANCR, a pGPH1 plasmid containing short hairpin (sh) RNA targeting DANCR (shDANCR), a pGPH1 plasmid containing scrambled control shRNA (shNC), pcDNA3.1-DANCR, and pcDNA3.1-NC were also obtained from GenePharma. Cells were transfected with the above vectors using the Lipofectamine 2000 transfection reagent (Invitrogen) as instructed by the manufacturer.

### Luciferase reporter assay

CTNNB1-3’UTR wild-type (WT) and CTNNB1-3’UTR mutant (MUT) sequences were inserted into the luciferase reporter vector pMIR-report. The potential binding sites between CTNNB1 and miR-320a were predicted by using the StarBase (http://starbase.sysu.edu.cn/index.php) and TargetScan (http://www.targetscan.org/vert_72/) web tools, whereas the targeted binding sites between CTNNB1 and DANCR were predicted by using the LncRNA2Target v2.0 (http://123.59.132.21/lncrna2target/index.jsp) and RNA Interactome Database (http://www.rna-society.org/raid/search.html) web tools. Mutations at the putative binding site (CTNNB1-3’UTR MUT_miR-320a, CTNNB1-3’UTR MUT_DANCR) were conducted and synthesized using a QuickChange site-directed mutagenesis Kit (Stratagene, California, USA). BMSCs were plated in a 96-well plate at 70% confluence and cotransfected with luciferase reporter vectors, miR-320a mimic, inhibitor oligos, or DANCR overexpression or shDANCR vectors using Lipofectamine 2000 (Invitrogen). After 48 h, the cells were lysed, and firefly luciferase activities were determined using the Dual-Luciferase Reporter Assay System (Promega, Madison, WI, USA) according to the manufacturer’s protocols. Firefly luciferase activity was normalized to Renilla activity.

### Animal models and grouping

Sixteen female C57BL/6 mice (8 weeks old, mean weight of 19 g) were purchased from SLAC Laboratory Animal Company, Ltd. (Shanghai, China) and assigned randomly into two groups: the bilateral ovariectomy group (OVX) and the sham operation control group. The mice were anesthetized with 5% ketamine and underwent bilateral ovariectomy (OVX; eight mice) or sham operation (sham; eight mice) in a bioclean environment. Skin incision was performed in both the OVX and sham groups, and the OVX group underwent ovary removal surgery. The bilateral ovaries from the sham group were identified and placed back into the abdominal cavity. The abdominal incisions were carefully sutured in both groups. The two groups of mice were housed separately with free access to food and drinking water for 12 weeks and prepared for subsequent experiments. The animal experimental procedures were approved in accordance with the Institutional Animal Care and Use Committee of Xiangya Hospital, Central South University (Changsha, Hunan, China). The mice were sacrificed 12 weeks after the operation, and dual-energy X-ray absorptiometry (Hologic, Bedford, MA, USA) was used to measure the femur bone density. Specifically, trabecular number (Tb.N) and trabecular thickness (Tb.Th) were quantified using micro-CT.

### Histological analysis by H&E and Alcian Blue staining

The femurs were dissected, fixed with 10% paraformaldehyde, decalcified with 14% ethylenediamine tetra-acetic acid and embedded in paraffin. To evaluate bone morphological changes, the samples were cut into coronal sections of 4 μm in thickness and stained with hematoxylin and eosin (H&E) or with Alcian Blue. Briefly, paraffin was removed from the paraffin-embedded bone samples, followed by three changes of xylene and rehydration in 100, 95, and 80% ethanol. The slides were stained with hematoxylin for 3 min and rinsed three times with deionized water for 30 s, followed by differentiation in 1% acid ethanol. The slides were then stained with 0.5% eosin for 30 s and dehydrated in ethanol, followed by three changes of xylene. A drop of neutral balsam mounting medium (Techyo, Shanghai) was added and spread well on the slide. The slide was naturally dried overnight and observed under a microscope (Leica DMIRB, Germany). Alcian Blue staining was performed using an Alcian Blue Stain Kit (Abcam, UK) according to the manufacturer’s instructions.

### Alkaline phosphatase (ALP) staining and activity detection

To evaluate osteogenic differentiation, human and mouse BMSCs were collected after 0, 7, and 14 days of culture in osteogenic differentiation induction medium. The ALP activity of the cell lysate was determined using an ALP colorimetric assay kit (BioVision, USA) according to the manufacturer’s protocol. In addition, histochemical ALP staining was conducted following the manufacturer’s instructions (GeFan Biotechnology, China).

### Mineral nodule quantification using Alizarin Red dye staining

BMSCs were cultured in osteogenic differentiation induction medium for two weeks, and the conditioned medium was changed every three days. To detect the calcified nodules, cells were fixed with 4% paraformaldehyde and stained with Alizarin Red (Sigma) for 30 min, as previously described^29^. The cells were washed three times in PBS and observed under an inverted microscope (Leica DMIRB, Germany).

### RNA extraction and qPCR detection

Total RNA of the tissue samples and cells was extracted using TRIzol (Invitrogen) according to the manufacturer’s protocols. RNA purity and concentration were assessed by UV spectrometry. Two micrograms of total RNA was reverse transcribed into cDNA using the PrimeScript RT reagent Kit (for mRNAs, Takara, Dalian, China) or TaqMan MicroRNA Reverse Transcription Kit (for miR-320a, Thermo Fisher Scientific, USA). The expression levels of DANCR, miR-320a, CTNNB1, TCF-1, RUNX2, ALP, osteopontin (OPN), and osteocalcin (OCN) were detected in an ABI 7500HT real‑time PCR system (Thermo Fisher Scientific) by the SYBR Premix EX Taq Kit (Takara). GAPDH and U6 small nuclear RNA (U6 snRNA) were used as internal references for mRNA and miRNA, respectively. The relative expression level was calculated by the 2^−ΔΔCt^ method. The primers for DANCR, miR-320a, CTNNB1, TCF-1, RUNX2, ALP, OPN, and OCN were synthesized by Sangon Biotech (Shanghai, China). The primer sequences used for qRT-PCR are listed in Table 1.

**Table 1:** Primers used for qRT-PCR.

Primer	Direction	Primer Sequence (5’—3’)
DANCR	Forward	CAGCTGACCCTTACCCTGAA
	Reverse	GACCCTGGGGTTGTTAGTCA
MiR-320a	Forward	GGGCTAAAAGCTGGGTTGA
	Reverse	CAGTGCGTGTCGTG GAGT
CTNNB1	Forward	CCCACTAATGTCCAGCGTTT
	Reverse	AACGCATGATAGCGTGTCTG
TCF-1	Forward	CTCAACCAGTCCCACCTGTC
	Reverse	CTCATCACCTGTGGGCTCTT
RUNX2	Forward	CGGAATGCCTCTGCTGTTAT
	Reverse	TTCCCGAGGTCCATCTACTG
ALP	Forward	AACCCCAGACCCTGAGTACC
	Reverse	CATGAGATGGGTCACAGACG
OPN	Forward	GATGGCCGAGGTGATAGTGT
	Reverse	GTGGGTTTCAGCACTCTGGT
OCN	Forward	GGCAGCGAGGTAGTGAAGAG
	Reverse	CTAGACCGGGCCGTAGAAG
U6	Forward	CTCGCTTCGGCAGCACA
	Reverse	AACGCTTCACGAATTTGCGT
GAPDH	Forward	CCAGGTGGTCTCCTCTGA
	Reverse	GCTGTAGCCAAATCGTTGT

### Western blot analysis

Western blot analysis was performed as described previously^30^. Cells were lysed in ice-cold RIPA lysis buffer, which was supplemented with protease inhibitor cocktail (Sigma). Nuclear proteins and cytoplasmic proteins were obtained for β-catenin detection using a Cell Nuclear and Cytoplasmic Protein Extraction Kit (Beyotime, China). A BCA protein assay kit (Beyotime) was then used to detect the concentration of the protein samples. Thirty micrograms of total protein was separated by 10% SDS-PAGE gels and then transferred to nitrocellulose membranes, followed by 1 h incubation with 5% nonfat milk blocking buffer. The membranes were incubated with primary antibodies against TCF-1 (1:1000 dilution, Abcam), RUNX2 (1:2000 dilution, Abcam), OCN (1:1000 dilution, Abcam), OPN (1:3000 dilution, Abcam), β-catenin (1:500 dilution, Abcam), and GAPDH (1:1000 dilution, Abcam) at 4 °C overnight. The membranes were washed three times in 0.1 M PBST and incubated with HRP-conjugated secondary antibodies (Abcam) for 1 h at room temperature. Bands were developed using chemiluminescence substance (Thermo Fisher Scientific). The proteins were quantified using Quantity One software (Bio-Rad Laboratories, Inc., USA).

### Statistical analysis

All experiments were conducted independently at least three times, with one representative experiment shown. Data were analyzed with Prism 6.0 (GraphPad Software, USA) and are expressed as the mean ± standard deviation (SD). Statistical evaluation was performed using Student’s *t*-test (two tailed) for comparisons between two groups or one-way analysis of variance (ANOVA) followed by Tukey’s post-hoc test for multiple comparisons. Spearman correlation analysis was performed to analyze the correlation between DANCR, miR-320a, and CTNNB1 in osteoporosis patients. A value of *P* < 0.05 was considered statistically significant for all analyses.

## Results

### The differential expression of DANCR, miR-320a, and CTNNB1 in osteoporosis patients

**Fig. 1:**
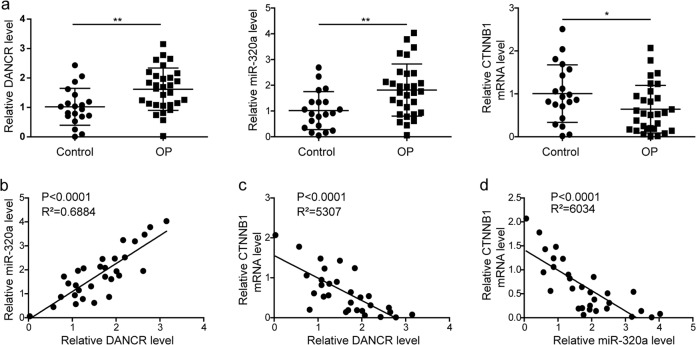
The differential expression of DANCR, miR-320a, and CTNNB1 in osteoporosis patients. **a** The expression levels of DANCR, miR-320a, and CTNNB1 were determined by qRT-PCR. **b** DANCR expression was positively correlated with miR-320a expression in BMSCs of osteoporosis patients (*n* = 30). **c**, **d** The expression of DANCR (**c**) and miR-320a (**d**) was negatively correlated with CTNNB1 expression in BMSCs of osteoporosis patients (*n* = 30). All experiments were conducted independently at least three times. *P < 0.05 and **P < 0.01.

To elucidate the biological functions of DANCR, miR-320a, and CTNNB1 in osteoporosis pathogenesis, we first verified and compared their expression levels in BMSCs derived from osteoporosis patients with those in control cells. We observed a relatively high expression of both DANCR and miR-320a and a remarkably lower expression of CTNNB1 in osteoporosis patients (Fig. 1a). We next performed coexpression analysis of these genes and found that the expression of miR-320a and DANCR was positively correlated (Fig. 1b), while DANCR and miR-320a expression exhibited a negative correlation with CTNNB1 expression (Fig. 1c, d). These results suggested that DANCR, miR-320a, and CTNNB1 might be related to the development of osteoporosis.

### The expression levels of DANCR, miR-320a, CTNNB1, and osteoblastic genes during osteogenic differentiation in vitro

**Fig. 2:**
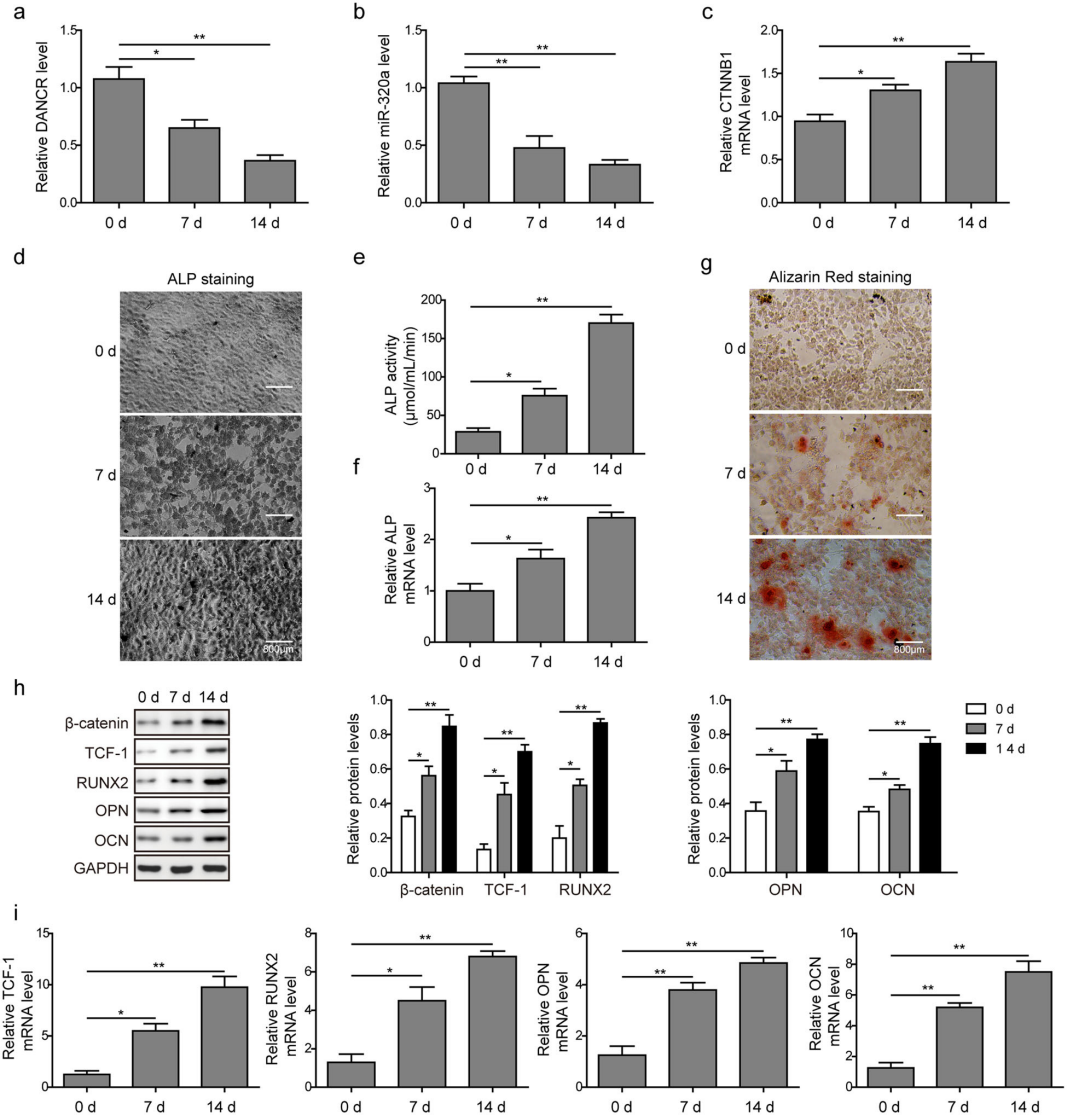
The expression levels of DANCR, miR-320a, CTNNB1, and osteoblastic genes during osteogenic differentiation in vitro. BMSCs were incubated in osteogenic induction medium for 0, 7, and 14 days, and the relative expression levels of **a** DANCR, **b** miR-320a, and **c** CTNNB1 were determined by qRT-PCR. **d** BMSCs were stained with ALP and observed under a microscope. **e**, **f** ALP activity (**e**) and ALP mRNA expression (**f**) were determined upon induction of osteoblast differentiation. **g** Cells were stained with Alizarin Red and observed under an inverted microscope. **h** Expression of β-catenin, TCF-1, RUNX2, OPN, and OCN at the protein level was determined by western blot on days 0, 7, and 14 after osteogenic induction. GAPDH was used as a loading control. **i** Relative mRNA levels of TCF-1, RUNX2, OPN, and OCN during osteogenic differentiation were determined by qRT-PCR. All experiments were conducted independently at least three times. *P < 0.05 and **P < 0.01.

We then examined whether the expression levels of DANCR, miR-320a, and CTNNB1 were altered after osteogenic differentiation induced in BMSCs. Interestingly, we observed that the expression levels of DANCR (Fig. 2a) and miR-320a (Fig. 2b) were gradually reduced, while CTNNB1 (Fig. 2c) was increased upon the induction of osteogenic differentiation. Accordingly, western blot analysis revealed increased expression of CTNNB1 at the protein level (β-catenin, Fig. 2h). Osteogenic differentiation was confirmed by ALP staining (Fig. 2d), ALP activity assay (Fig. 2e), ALP mRNA level (Fig. 2f), and Alizarin Red staining (Fig. 2g). We next investigated the expression of TCF-1, RUNX2, OCN, and OPN during osteogenic differentiation. Western blot analysis showed that the protein levels of TCF-1, RUNX2, OPN, and OCN were significantly increased in BMSCs at 7 and 14 days of osteogenic differentiation (Fig. 2h). In addition, the mRNA levels of TCF-1, RUNX2, OPN, and OCN also increased over time (Fig. 2i).

### CTNNB1 was directly regulated by miR-320a during osteogenic differentiation

**Fig. 3:**
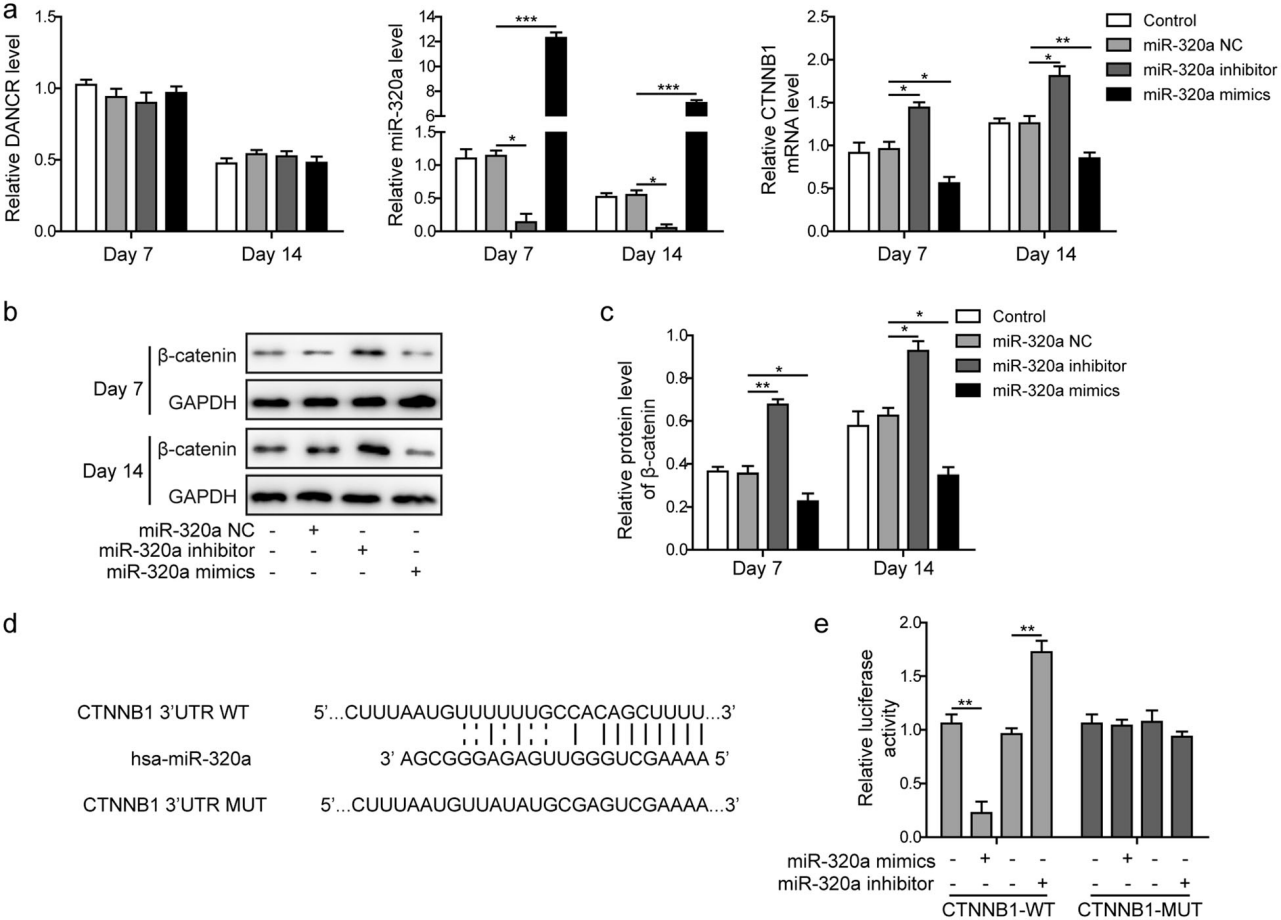
CTNNB1 was directly regulated by miR-320a during osteogenic differentiation. **a** Relative expression levels of DANCR, miR-320a, and CTNNB1 were assessed by qRT-PCR. **b** The impact of miR-320a on β-catenin expression was verified by western blot analysis. **c** Quantitative analysis of β-catenin protein expression in **b**. **d** The potential binding sites between *CTNNB1* and miR-320a were identified using StarBase and TargetScan web tools, and a corresponding mutant construct (CTNNB1-MUT) with the indicated sequence was generated. **e** CTNNB1 wild-type (CTNNB1-WT) and CTNNB1-MUT were cloned into a luciferase reporter vector, and BMSCs were cotransfected with lentivirus encoding miR-320a inhibitor or miR-320a mimics. All experiments were conducted independently at least three times. *P < 0.05, **P < 0.01, and ***P < 0.001.

To investigate the regulatory effects of miR-320a on CTNNB1, we infected BMSCs with lentivirus bearing miR-320a inhibitor or mimic oligos. Cells were subsequently maintained in osteogenic differentiation medium for 7 or 14 days. We found in the qRT-PCR analysis that CTNNB1 mRNA expression was significantly inhibited by miR-320a mimics and promoted by miR-320a inhibitor, while DANCR expression remained unchanged upon infection (Fig. 3a). Western blot assays further demonstrated that miR-320a overexpression resulted in the decreased expression of CTNNB1 at the protein level (β-catenin), whereas miR-320a inhibition promoted CTNNB1 expression (Fig. 3b, c). To test the targeting of CTNNB1 by miR-320a, we predicted the potential binding sites using the StarBase and TargetScan web tools. As shown in Fig. 3d, an 11-nucleotide base pairing was identified between CTNNB1 and miR-320a. We next performed a luciferase reporter assay to validate the complementary binding above (Fig. 3e). Consistent with our prediction, the luciferase activity of CTNNB1-WT was significantly repressed by miR-320a overexpression and enhanced by the miR-320a inhibitor. Mutations of the predicted binding sites completely abrogated the downregulation of CTNNB1 by miR-320a.

### MiR-320a downregulated osteogenic differentiation and inhibited the β-catenin signaling pathway

**Fig. 4:**
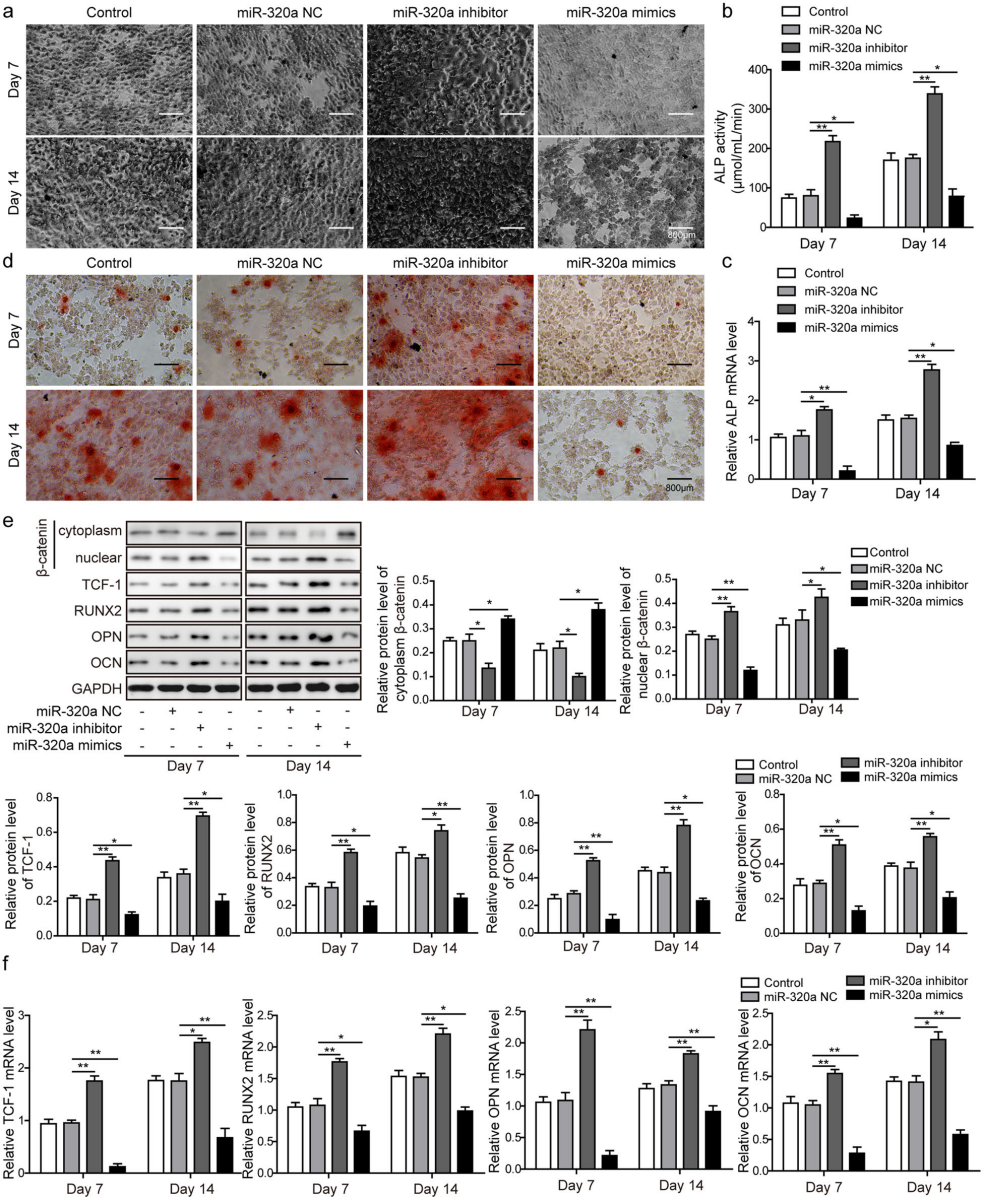
MiR-320a downregulated osteogenic differentiation and inhibited the β-catenin signaling pathway. Osteogenic differentiation was evaluated at days 7 and 14 by staining the cells with **a** ALP or **d** Alizarin Red. The cells were observed under an inverted microscope. **b** The staining results were further confirmed by ALP activity quantification. **c** qRT-PCR was performed to evaluate the mRNA expression of ALP. **e** Western blotting was performed to evaluate the expression of β-catenin in the cytoplasm and nucleus, TCF-1, RUNX2, OPN, and OCN. **f** Relative mRNA levels of TCF-1, RUNX2, OPN, and OCN upon lentiviral infection were determined by qRT-PCR. BMSCs were infected with lentivirus encoding miR-320a inhibitor or mimics or with empty viruses, and cells were collected and lysed at day 7 and day 14. All experiments were conducted independently at least three times. *P < 0.05 and **P < 0.01.

To determine whether miR-320a was involved in osteogenic differentiation regulation, we transfected BMSCs with lentivirus encoding miR-320a inhibitor or mimics and evaluated cellular osteogenic differentiation by ALP staining (Fig. 4a), ALP activity assay (Fig. 4b), ALP mRNA level (Fig. 4c) and Alizarin Red staining (Fig. 4d). We demonstrated by several methods that ALP levels were elevated in BMSCs expressing the miR-320a inhibitor compared with the control cells, and BMSCs with miR-320 overexpression showed a trend in the opposite direction. Consistent with the ALP-related results, Alizarin Red staining indicated that the miR-320a inhibitor group formed the most mineralized nodules, whereas little or no mineralized nodules were observed in the miR-320a mimics group (Fig. 4d). To investigate the biological role of miR-320a in osteogenic differentiation, we analyzed the protein and mRNA levels of osteogenic markers in BMSCs transfected with miR-320a inhibitor or mimics. Western blot results showed that miR-320a markedly inhibited the expression of TCF-1, RUNX2, OPN, and OCN at 7 and 14 days after osteogenic differentiation induction. The protein levels of these osteogenic markers were greatly promoted by miR-320a inhibition (Fig. 4e). Notably, the expression level of β-catenin in the nucleus was clearly increased by miR-320a inhibition but decreased upon miR-320a overexpression. However, the expression level of β-catenin in the cytoplasm showed the opposite trend (Fig. 4e). Similarly, inhibition of miR-320a expression increased the mRNA levels of TCF-1, RUNX2, OPN, and OCN in BMSCs, which were decreased by miR-320a overexpression (Fig. 4f).

### The effect of the miR-320a inhibitor on CTNNB1 was suppressed by DANCR overexpression

**Fig. 5:**
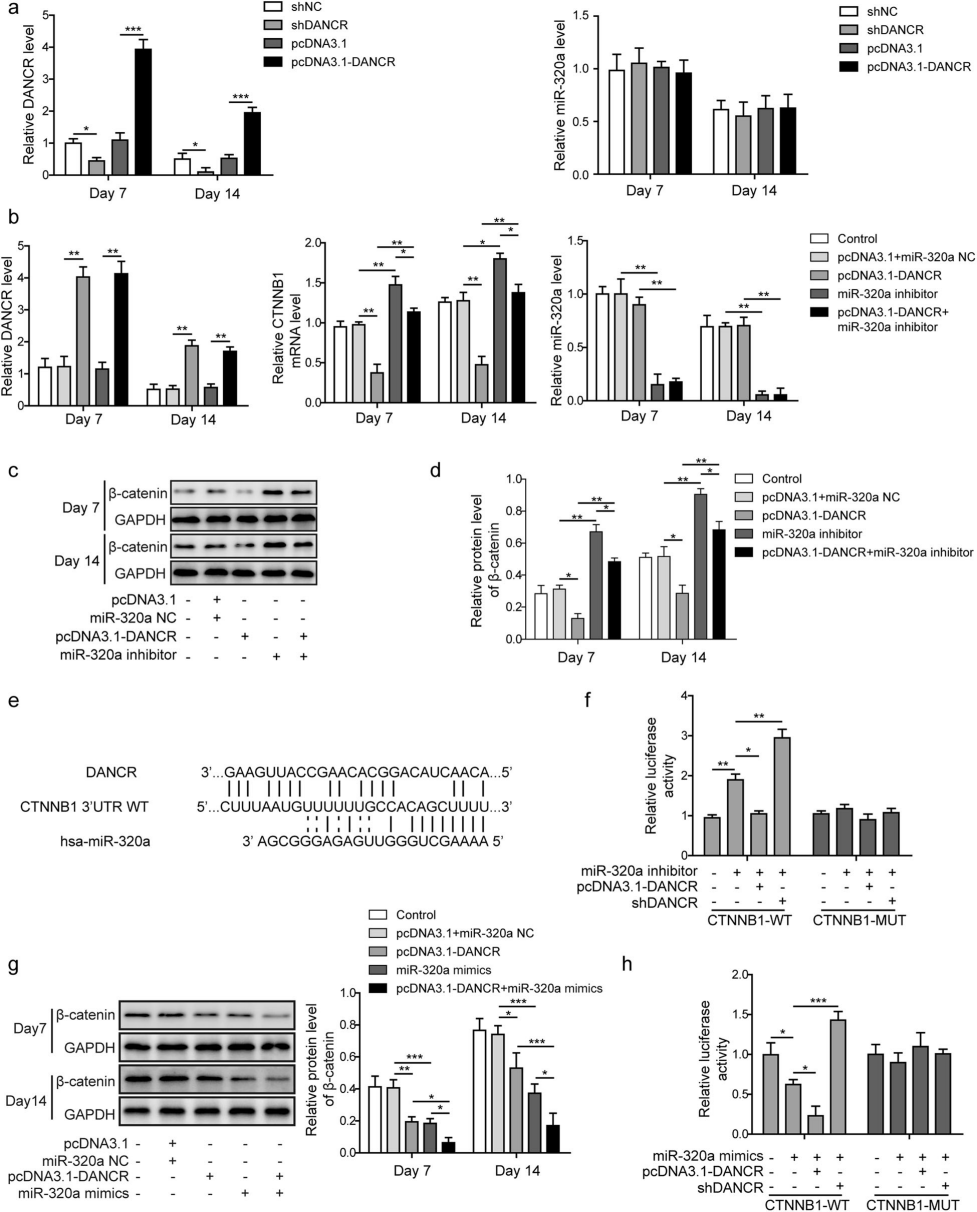
The effect of the miR-320a inhibitor on CTNNB1 was suppressed by DANCR overexpression. **a** Relative expression levels of DANCR and miR-320a were measured by qRT-PCR when DANCR was overexpressed or silenced. **b** Relative expression levels of DANCR, miR-320a, and CTNNB1 were detected by qRT-PCR. (**c**) Western blot analysis was performed to determine the protein expression of CTNNB1 (β-catenin). **d** Quantitative analysis of β-catenin protein expression in **c**. **e** The binding sites of CTNNB1 with DANCR or miR-320a were predicted using LncRNA2Target v2.0 and RNA Interactome Database web tools. **f** A luciferase reporter assay was performed to confirm the binding of CTNNB1 with DANCR and miR-320a. CTNNB1-WT and CTNNB1-MUT were cloned into a luciferase reporter vector, and BMSCs were cotransfected with lentivirus encoding miR-320a inhibitor, DANCR overexpression or knockdown vectors. **g** BMSCs were transfected with pcDNA3.1-DANCR, miR-320a mimic-carrying lentivirus or negative controls. β-Catenin protein expression was determined by western blot. **h** Relative luciferase activity of CTNNB1-WT and CTNNB1-MUT was measured upon manipulation of the expression level of DANCR or miR-320a. All experiments were conducted independently at least three times. *P < 0.05, **P < 0.01, and ***P < 0.001.

To validate the involvement of DANCR in osteoporosis, which we observed in the qRT-PCR results, after determining the DANCR overexpression or knockdown transfection efficiency in BMSCs, we found that the expression level of miR-320a was not affected by DANCR overexpression or knockdown (Fig. 5a). However, the relative mRNA level of CTNNB1 was remarkably inhibited by DANCR. We observed that DANCR overexpression abolished the promoting effect of the miR-320a inhibitor on CTNNB1 mRNA expression (Fig. 5b). Similarly, western blot analysis showed that DANCR overexpression decreased the CTNNB1 protein level (β-catenin) in BMSCs by almost half at 7 and 14 days of osteogenic differentiation. Furthermore, the elevated β-catenin expression in BMSCs by miR-320a knockdown was counteracted by DANCR cotransfection (Fig. 5c, d).

Next, we aligned RNA sequences and identified the putative binding sites of the 3’-UTR of CTNNB1 with DANCR and miR-320a using the LncRNA2Target v2.0 and RNA Interactome Database web tools. As shown in Fig. 5e, a 16-nucleotide base pairing was identified between CTNNB1 and DANCR. We next performed a luciferase reporter assay in BMSCs to validate the complementary binding above (Fig. 5f). The relative luciferase activity of the CTNNB1 reporter was greatly enhanced by the miR-320a inhibitor and reduced by DANCR cotransfection compared with that in the control cells. Accordingly, DANCR knockdown further boosted the promoting effect of the miR-320a inhibitor on the relative luciferase activity of the CTNNB1 reporter. We next performed western blot and luciferase reporter assays and evaluated whether CTNNB1 was affected by DANCR and miR-320a overexpression. Interestingly, we observed that overexpression of DANCR or miR-320a both decreased β-catenin expression and the luciferase activity of CTNNB1-WT, and they had an additive effect (Fig. 5g, h). Furthermore, knockdown of DANCR rescued the decrease in luciferase activity of CTNNB1-WT caused by miR-320a mimics. The effect of DANCR and miR-320a mimics on the luciferase activity of the CTNNB1 promoter was completely abolished by site-directed mutagenesis of the binding sites (Fig. 5h).

### DANCR attenuated the osteogenic differentiation and deactivated the β-catenin signaling pathway induced by the miR-320a inhibitor in BMSCs

**Fig. 6:**
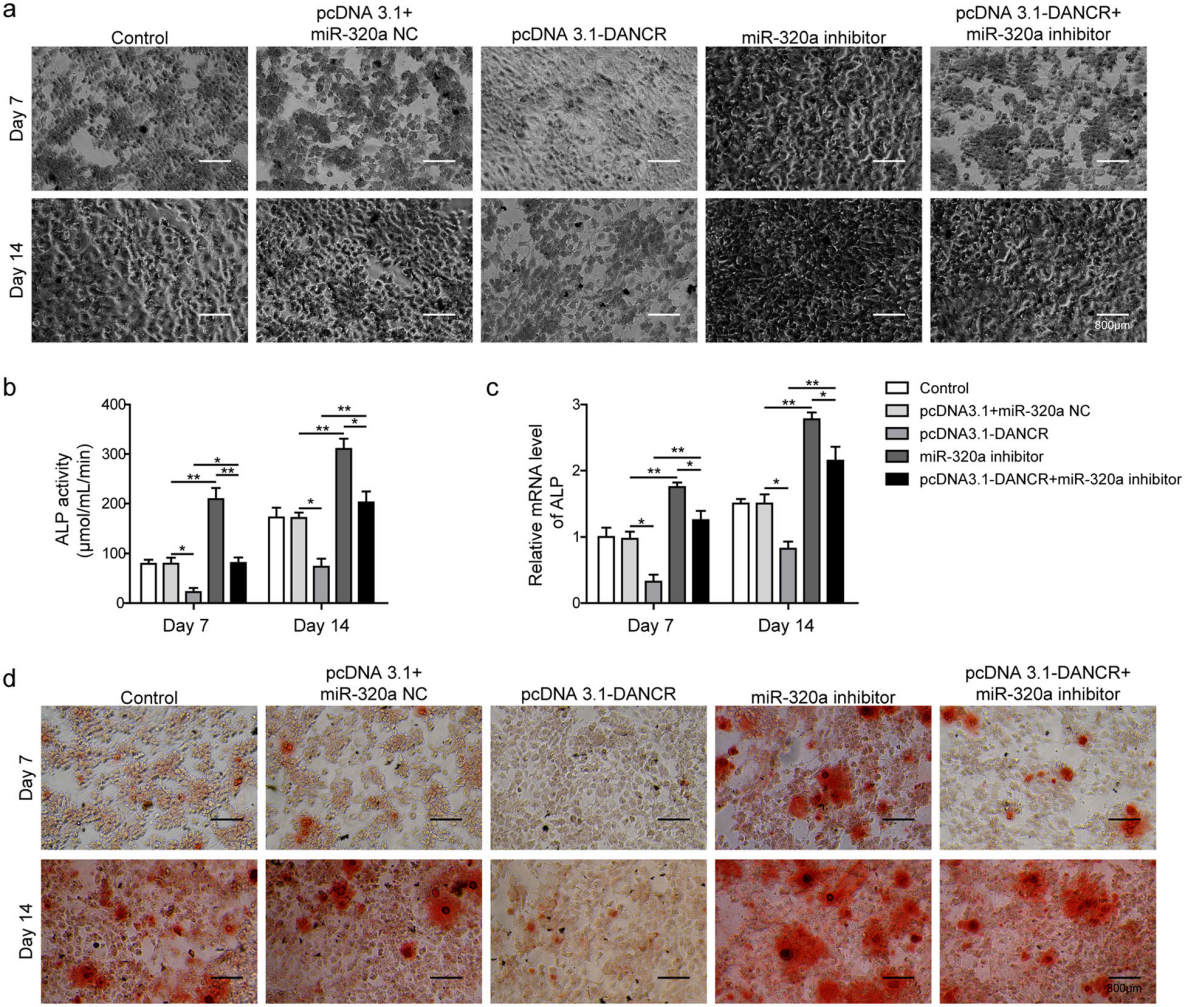
DANCR overexpression attenuated the osteogenic differentiation induced by the miR-320a inhibitor. BMSCs were infected with lentivirus encoding miR-320a inhibitor and/or DANCR overexpression vector, followed by incubation in osteogenic differentiation induction medium for 7 or 14 days. Cells were subsequently stained with **a** ALP and **d** Alizarin Red and observed under an inverted microscope. **b** Cells were lysed, and the relative ALP activity was detected. **c** Relative mRNA expression of ALP was determined by qRT-PCR. All experiments were conducted independently at least three times. *P < 0.05 and **P < 0.01.

After demonstrating the inhibitory effect of DANCR on miR-320a-regulated CTNNB1 expression, we further verified the impact of DANCR on osteogenic differentiation induced by the miR-320a inhibitor. ALP and Alizarin Red staining showed that DANCR overexpression inhibited BMSC osteogenic differentiation and effectively decreased mineral nodule formation (Fig. 6a, d) at 7 and 14 days compared with that in the control cells. Intriguingly, cotransfection of DANCR largely neutralized the boosting effect of miR-320a knockdown on BMSC osteogenic differentiation and mineralized nodule formation. Consistent with the staining results, we observed complete inhibition of ALP levels by DANCR overexpression in ALP activity quantification and qRT-PCR assays compared with that in the control cells (Fig. 6b, c).

**Fig. 7:**
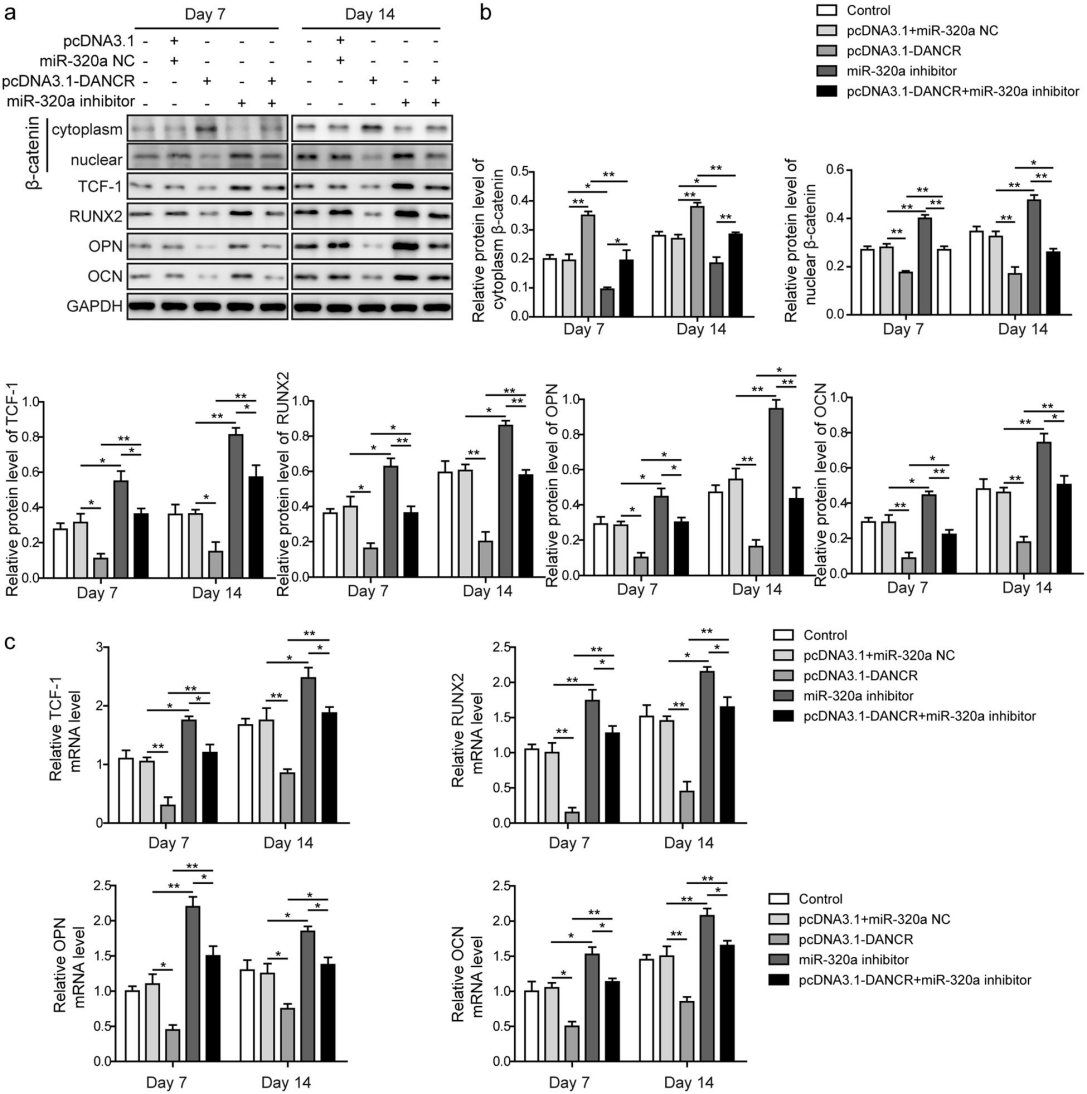
DANCR overexpression suppressed the promoting effect of the miR-320a inhibitor on the β-catenin signaling pathway. BMSCs were infected with lentivirus encoding miR-320a inhibitor and/or DANCR overexpression vector and further incubated in osteogenic differentiation inducing medium for 7 or 14 days. **a** Cells were lysed, and western blot analysis was performed to analyze the protein expression of β-catenin in the cytoplasm and nucleus, TCF-1, RUNX2, OPN, and OCN. **b** Quantitative analysis of protein expression in **a**. **c** Relative mRNA levels of TCF-1, RUNX2, OPN, and OCN were evaluated by qRT-PCR. All experiments were conducted independently at least three times. *P < 0.05 and **P < 0.01.

We next investigated whether DANCR affected the β-catenin signaling pathway, which was activated by miR-320a knockdown, and analyzed the expression of osteogenic differentiation markers at the protein (Fig. 7a, b) and mRNA levels (Fig. 7c). As shown in Fig. 7a and b, the protein expression of β-catenin in the nucleus was significantly reduced, while its expression in the cytoplasm was increased in the DANCR overexpression plus miR-320a inhibitor group compared with the miR-320a inhibitor only group, suggesting that DANCR suppressed the β-catenin signaling pathway, which was activated by the miR-320a inhibitor. Additionally, the protein levels of osteogenic differentiation markers showed the same trend as nuclear β-catenin. qRT-PCR results showed similar expression patterns of the osteogenic markers in BMSCs (Fig. 7c).

### DANCR, miR-320a, and CTNNB1 were differentially expressed in the osteoporosis mouse model

**Fig. 8:**
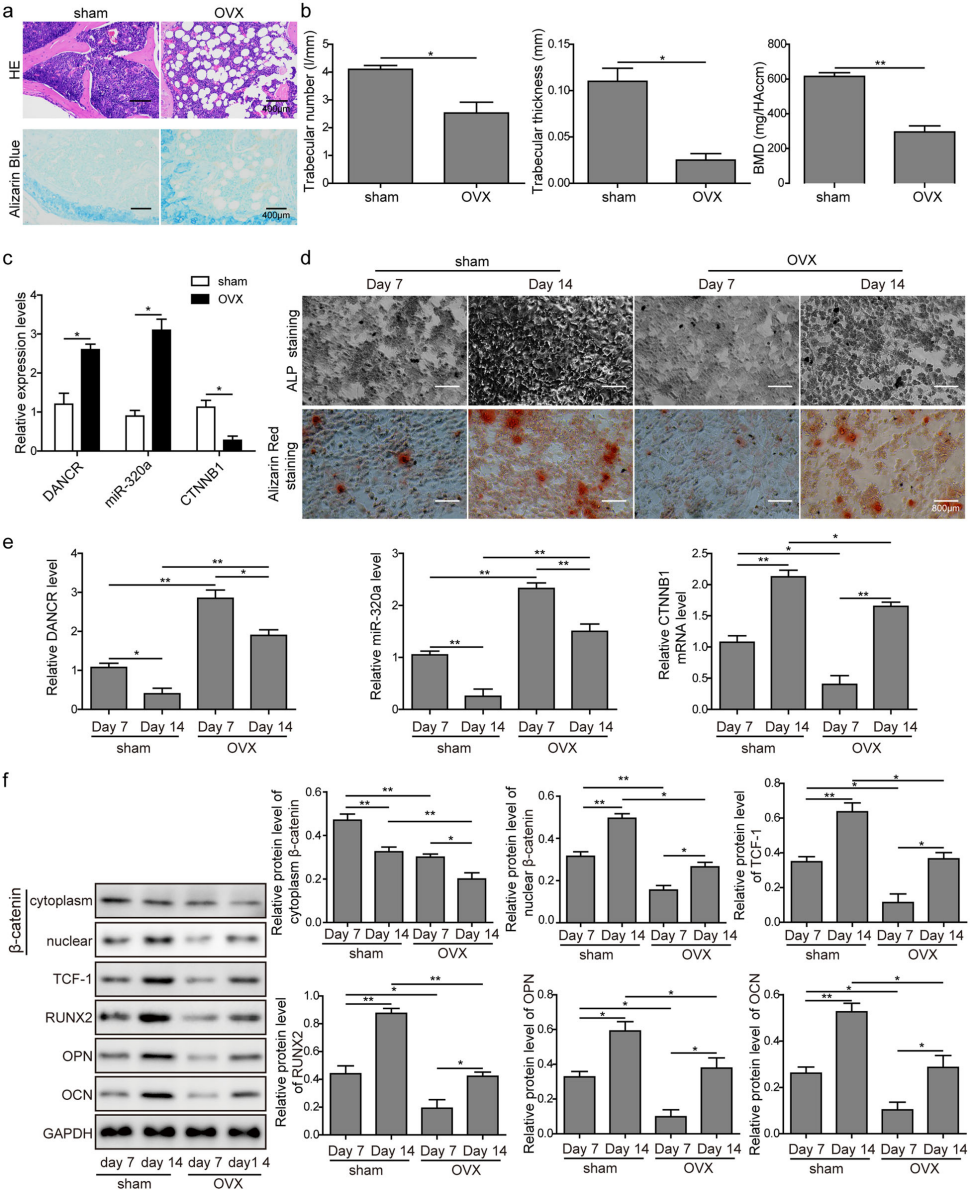
DANCR, miR-320a, and CTNNB1 were differentially expressed in the osteoporosis mouse model. **a** Bone tissues from the OVX and sham groups were extracted and stained with H&E and Alcian Blue. **b** Tb.N, Tb.Th, and BMD were measured on micro-CT and dual-energy X-ray absorptiometry, respectively. **c** Relative expression levels of DANCR, miR-320a, and CTNNB1 in mouse bones were determined by qRT-PCR. **d** BMSCs from femurs and tibias in both the sham and OVX groups were isolated and cultured in osteogenic differentiation induction medium for 7 and 14 days. Cells were stained with ALP or Alizarin Red. **e** Cells after osteogenic differentiation induction were lysed, and qRT-PCR was performed to compare the relative expression levels of DANCR, miR-320a, and CTNNB1 in the OVX and sham groups. **f** Western blot analysis was performed to evaluate the protein expression of β-catenin in the cytoplasm and nucleus, TCF-1, RUNX2, OPN, and OCN of mouse BMSCs upon 7 and 14 days of osteogenic differentiation induction. All experiments were conducted independently at least three times. *P < 0.05 and **P < 0.01.

Finally, we attempted to elucidate the biological functions of DANCR, miR-320a, and CTNNB1 in osteoporotic mice. For this purpose, we first dissected the femurs of sacrificed mice and stained them with HE or Alcian Blue. Microscopic views of the staining results revealed a reduced subchondral trabecular bone volume in the OVX group (Fig. 8a). In addition, micro-CT and bone densitometry data demonstrated that Tb.N, Tb.Th, and BMD were significantly decreased in OVX mice compared with the sham group (Fig. 8b). In addition, we verified the altered expression of DANCR, miR-320a, and CTNNB1 in bone samples derived from osteoporotic mice. Indeed, qRT-PCR results showed that the expression levels of DANCR and miR-320a in OVX mice were markedly higher than those in the sham group, while the relative mRNA level of CTNNB1 was dramatically reduced (Fig. 8c).

We next isolated BMSCs from mouse femurs and tibias and cultured them in osteogenic differentiation induction medium for 7 or 14 days. ALP and Alizarin Red staining experiments showed that the processes of osteogenic differentiation and mineralized nodule formation were inhibited in BMSCs isolated from OVX mice (Fig. 8d). Additionally, we observed that upon osteogenic differentiation induction, DANCR and miR-320a were upregulated in the OVX group, whereas CTNNB1 was downregulated compared to their levels in the sham group (Fig. 8e). In accordance with the osteogenic differentiation and mineralization results, we observed lower protein expression of TCF-1, RUNX2, OPN, and OCN in OVX mice than in the sham group (Fig. 8f). Additionally, we found that the protein level of β-catenin in the cytoplasm was reduced, while its expression in the nucleus was significantly increased after osteogenic differentiation in both the sham and OVX groups (Fig. 8f).

## Discussion

Osteoporosis is one of the most common skeletal diseases and causes millions of bone fractures yearly. The annual cost of osteoporosis, including pharmacological interventions and hospitalizations, is estimated at billions of dollars worldwide^31^. An imbalance between the bone resorption and formation processes is the main cause of osteoporosis. Although numerous treatment methods exist, concerns have arisen regarding adverse side effects and the long-term efficacy of the currently available palliative medications. Furthermore, the development of curative osteoporosis therapeutics is impeded by our limited knowledge of the underlying mechanisms, especially how the transcriptional networks and signaling pathways are tightly coordinated at the genetic level during bone development. Since numerous factors are known to result in osteoporosis and the molecular basis underlying the pathological progression might largely differ among different osteoporosis conditions, in the present study, we focused on postmenopausal osteoporosis, the most common type of osteoporosis. Here, we applied in vitro and in vivo methods to investigate the effect of the interactions between DANCR and miR-320a on CTNNB1 molecular expression in osteogenic differentiation and revealed their synergistic regulation of the Wnt/β-catenin signaling pathway.

Accumulating evidence indicates that noncoding RNAs, including miRNAs and lncRNAs, play important roles in osteoclastogenesis and osteoblast differentiation^18,32^, and their dysregulated expression is observed in various bone diseases^23,33,34^. Among them, miR-320a recently gained increasing interest due to its differential expression in osteoporotic vs osteoarthritic samples^35^. In this study, we isolated BMSCs from osteoporosis patients and found that the molecular levels of DANCR and miR-320a were remarkably higher than those in healthy control cells, whereas CTNNB1 expression was downregulated. Furthermore, we reported for the first time that DANCR expression was positively correlated with the expression of miR-320a during osteoporosis development and that CTNNB1 expression was negatively correlated with the expression of DANCR and miR-320a. Our qRT-PCR and western blot results showed that the expression levels of DANCR and miR-320a were attenuated in BMSCs during the osteogenic differentiation process, while the mRNA and protein levels of CTNNB1 were increased. Our results are consistent with previous reports showing that CTNNB1 is downregulated in samples from osteoporosis patients^36^, that miR-320a is overexpressed in human osteoporotic bone tissues^24,37^ and that downregulation of DANCR can promote osteogenic differentiation^38,39^. However, we are the first to explore the correlation between DANCR, miR-320a, and CTNNB1 in osteogenic differentiation in osteoporosis.

The Wnt/β-catenin signaling pathway plays a crucial role in bone homeostasis by stimulating osteoblast generation and decreasing osteoclast differentiation, and it was demonstrated that osteogenic differentiation was reduced by the inhibition of the Wnt/β-catenin signaling pathway^17,40^. OPN and OCN are biomarkers of terminally differentiated osteoblasts, and their expression is promoted by the transcription factors TCF-1 and RUNX2^41^. It was reported that the expression of RUNX2 and TCF-1 was regulated by the activity of Wnt/β-catenin signaling^42^. In the present study, we demonstrated for the first time that silencing miR-320a activated the Wnt/β-catenin signaling pathway by promoting the accumulation of nuclear β-catenin in BMSCs and accelerated osteogenic mineralization, as indicated by the enhanced expression levels of the abovementioned osteogenic differentiation markers. Previous evidence suggests that miR-320a suppresses CTNNB1 expression and regulates the Wnt/β-catenin signaling pathway in various diseases^25,43,44^. Our luciferase reporter assays demonstrated for the first time that CTNNB1 was the direct downstream target of miR-320a in the osteogenic differentiation process and that miR-320a inhibited CTNNB1 expression at both the mRNA and protein levels.

Interestingly, a recent study and bioinformatic prediction reported the suppression of CTNNB1 by DANCR in hepatocellular carcinoma^45^. In the present study, we validated the interactions between CTNNB1 and miR-320a or DANCR by performing luciferase reporter assays and demonstrated that DANCR overexpression in BMSCs abolished the effects of the miR-320a inhibitor on osteogenic differentiation and β-catenin signaling pathway activation. Although the expression levels of DANCR and miR-320a were not affected by each other, overexpression of DANCR or miR-320a both decreased β-catenin expression and the luciferase activity of CTNNB1-WT, and they exhibited an additive effect. It was previously reported that DANCR was upregulated in blood mononuclear cells from osteoporosis patients and increased bone resorption activity by secreting osteoclastogenic factors such as IL-6 and TNF-α^23^. Accordingly, we proved in the present study that DANCR overexpression was positively correlated with osteoporosis and efficiently inhibited osteogenic differentiation in BMSCs. Moreover, our *in vivo* experiments confirmed that OVX mice had significantly decreased bone density, trabecular number and trabecular thickness. Consistent with our in vitro experiments, we reported in the present study that DANCR and miR-320a were overexpressed and CTNNB1 was expressed at low levels in our osteoporosis mouse model, leading to the inhibition of the β-catenin signaling pathway.

Our data, however, did not reveal the upstream regulatory network of DANCR and miR-320a, and the possible molecular mechanisms remain elusive. Furthermore, we would need to thoroughly investigate the direct impacts of the molecules mentioned above, since no interventional studies were performed at the animal level in our present work. Our clinical research was thus far limited to determining the expression of these key factors, whereas no experiments were conducted for mechanism verification. Finally, our present work restricted the range of clinical samples to female postmenopausal osteoporosis patients and performed animal experiments on ovariectomized mice. For a broader study of osteoporosis physiopathology, we will need to enlarge our patient range and generate different osteoporosis animal models in the future.

Overall, we identified for the first time aberrant expression of DANCR, miR-320a, and CTNNB1 in osteoporosis patients and an OVX animal model. Moreover, we revealed that osteogenic differentiation in BMSCs was regulated by a novel genetic network that consisted of DANCR, miR-320a, and CTNNB1 through the Wnt/β-catenin signaling pathway. Notably, miR-320a negatively regulated osteogenic differentiation in BMSCs by directly targeting CTNNB1 and inhibiting the Wnt/β-catenin signaling pathway. The regulatory effect of the miR-320a inhibitor on CTNNB1 was abolished by DANCR. However, further efforts are needed to investigate whether and how other molecules interact with DANCR, miR-320a, and CTNNB1 during osteoporosis development, and the important question regarding the reason for DANCR upregulation in osteoporosis patients remains unanswered. This validated regulatory network could provide potential candidates for diagnostic markers or therapeutic targets for osteoporosis. Therefore, our results provide not only unique insight into osteoporosis pathogenesis but may also aid in the development of specific and effective therapeutic tools for osteoporosis.

## Data Availability

All data generated or analyzed during this study are included in this published article.
